# Multiple criteria decision analysis approach to consider therapeutic innovations in the emergency department: The methoxyflurane organizational impact in acute trauma pain

**DOI:** 10.1371/journal.pone.0231571

**Published:** 2020-04-15

**Authors:** Virginie Eve Lvovschi, Maxime Maignan, Karim Tazarourte, Mohamed Lamine Diallo, Caroline Hadjadj-Baillot, Nathalie Pons-Kerjean, Frederic Lapostolle, Claude Dussart

**Affiliations:** 1 Emergency Department, Rouen University Hospital, Normandie Univ, UNIROUEN, INSERM U1073, Rouen, France; 2 Emergency Department, Grenoble University Hospital, Univ. Grenoble Alpes, INSERM U1042, CHU Grenoble Alpes, HP2, Grenoble Alps University, Grenoble, France; 3 Emergency Department, Edouard Herriot Hospital, Lyon Public Hospices, Lyon, France; Health Services and Performance Research, HESPER, EA, Claude Bernard University, Lyon, France; 4 Pharmacy Department, Grand Hôpital de l’Est Francilien, Meaux, France; 5 Pharmacy Department, CHU de Bordeaux, Bordeaux, France; 6 Pharmacy Department, Beaujon Hospital, APHP, Clichy, France; 7 SAMU 93, Avicenne Hospital-APHP, Bobigny, France; INSERM U942, Paris 13 University, Paris, France; 8 Lyon Public Hospices, Central Pharmacy, Lyon, France; EA, Systemic Health Pathway Laboratory, University Claude Bernard, Lyon, France; Technion - Israel Institute of Technology, ISRAEL

## Abstract

**Background:**

Acute trauma pain is poorly managed in the emergency department (ED). The reasons are partly organizational: ED crowding and rare trauma care pathways contribute to oligoanalgesia. Anticipating the organizational impact of an innovative care procedure might facilitate the decision-making process and help to optimize pain management.

**Methods:**

We used a multiple criteria decision analysis (MCDA) approach to consider the organizational impact of methoxyflurane (self-administered) in the ED, introduced alone or supported by a trauma care pathway. A MCDA experiment was designed for this specific context, 8 experts in emergency trauma care pathways (leading physicians and pharmacists working in French urban tertiary hospitals) were recruited. The study involved four steps: (i) Selection of organizational criteria for evaluating the innovation’s impact; (ii) assessment of the relative weight of each criterion; (iii) choice of appropriate scenarios for exploring the organizational impact of MEOX under various contexts; and (iv) software-assisted simulation based on pairwise comparisons of the scenarios. The final outcome measure was the expected overall organizational impact of methoxyflurane on a 0-to-100 scale (score >50: positive impact).

**Results:**

Nine organizational criteria were selected. "Mean length of stay in the ED" was the most weighted. Methoxyflurane alone obtained 59 as a total score, with a putative positive impact for eight criteria, and a neutral effect on one. When a trauma care pathway was introduced concomitantly, the impact of methoxyflurane was greater overall (score: 75) and for each individual criterion.

**Conclusions:**

Our model highlighted the putative positive organizational impact of methoxyflurane in the ED—particularly when supported by a trauma care pathway—and the relevance of expert consensus in this particular pharmacoeconomic context. The MCDA approach could be extended to other research fields and healthcare challenges in emergency medicine.

## Introduction

In the emergency department (ED), acute trauma pain is not always managed optimally [1]. The causes of this failure (referred to as oligoanalgesia) are well known; the primary reason is ED crowding, which is accentuated by poor organization in treatment zones [2–4]. The growing prevalence of visits to the ED observed in most Western countries increases the risk of ED crowding, which in turn raises the likelihood of negative clinical outcomes [5, 6].

The implementation of integrated care pathways (as a form of guideline-based care) is an organizational response to changes in patient flows, and is recommended in the current guidelines [7]. Care provision is based on the recognition of a particular patient profile and then the application of an appropriate decision tree for diagnosis and treatment. Furthermore, the introduction of an innovative care procedure will have an impact on the host organization, and can sometimes even prompt the specific development of new sectors. The recent example of changes in stroke management is a reminder of how this evolution is inevitable for each therapeutic innovation: the advent of thrombectomy prompted physicians to redefine their protocols for neurovascular care in the ED [8]. There may be a gap between how work is currently organized and how it should be organized after the introduction of innovative care. The analysis of organizational impacts is therefore mainly based on process analysis.

The relevance of an organizational structure is often evaluated concomitantly with the quality of care, using various indicators [9]. In the ED, pain relief is a key indicator. In fact, pain is the most common reason for attending the ED. Around 50% of the patients admitted to the ED (and 80% of the patients with trauma pain) suffer from intense pain [10, 11].

Although powerful analgesics (such as morphine) can be used to effectively treat severe acute trauma pain, this option has proved to be unsatisfactory on the organizational level. In fact, the use of morphine is scrupulously restricted, in order to guarantee its effectiveness and safety and to limit misuse [12]. Furthermore, the optimal use of morphine in the ED requires titration (i.e. bolus injection until relief is achieved), which requires venous access and the availability of personnel for reassessing the pain every five minutes [13]. In addition, morphine’s potential side effects mean that close clinical monitoring is required. The use of morphine under non-optimal conditions (e.g. ED crowding) is therefore not always easy or preferable. Unfortunately, alternative treatment options (other drug classes or other administration routes—even non-invasive ones) are not appropriate in all situations, and may not be sufficiently effective.

In the complex organizational context of emergency medicine, it therefore appears that the choice of an analgesic strategy cannot be exclusively based on clinical and health economics criteria–the criteria most commonly used to consider innovations in healthcare [14, 15]. Importantly, failure to take account of a potential organizational impact will bias the assessment of a healthcare innovation, and will mean that (i) care resources as a whole are not optimized, and (ii) the cost/benefit ratio could disadvantage the institution. Hence, the organizational impact of an innovation now appears to be another key factor in decision-making [16]. Accordingly, the recent interest in this new factor had created a need for high-performance decision support tools as part of a systemic approach to organizational issues in health economics. A technique known as multiple criteria decision analysis (MCDA) addresses this need [17]. Many hospital departments are now applying MCDA to their management of innovations in care [18–20].

Multiple criteria decision analysis is a standardized method; the International Society for Pharmacoeconomics and Outcomes Research has issued good practice guidelines on the implementation of MCDA by healthcare professionals. In essence, MCDA enables the pairwise comparison of organizations or systems [17]. It also facilitates the identification of the optimal solution when the decision-making process is complex, regardless of the final objective. For example, one can compare the likely impact of a new therapeutic strategy in a medical department with that in a surgical department.

We reasoned that MCDA would be a relevant method for facilitating the choice of a pain management strategy for a given organizational mode in the ED. In fact, MCDA can (i) identify and formulate a decision-making problem in an imprecise or unstable environment, and thus (ii) provide insight into complex issues by testing several possible organizational options.

Deciding on a pain management strategy in the ED requires a set of often heterogeneous factors to be taken in account: differences in care teams, seasonality (by day, week or year), the disease patterns encountered, etc. Multiple criteria decision analysis not only provides a useful systemic approach but also facilitates multidisciplinary working by stimulating dialogue between healthcare professionals and emphasizing the shared, reasoned nature of the decision-making process. This method is relatively easy to apply and can easily be adopted by physicians or other healthcare professionals (pharmacists, nurses, etc.) without specific theoretical or statistical knowledge. It can be used with a panel of experts, and does not necessarily require exhaustive participation (i.e. all the healthcare professionals in a hospital department).

Methoxyflurane (MEOF) delivered by a Penthrox^®^ inhaler is a self-administered, inhaled analgesic indicated in the treatment of moderate-to-severe acute trauma pain [21, 22]. Several clinical studies have emphasized MEOF’s efficacy, rapid onset of action, and value in pain self-management [21, 23–25]. However, the organizational impact of including MEOF in the therapeutic arsenal for analgesia in the ED has not previously been studied. As would be the case for any other therapeutic innovation in the ED, the absence of this type of evaluation calls MEOF’s pharmacoeconomic relevance into question. In recent years, trauma care pathways have been considerably diversified and optimized for the treatment of life-threatening injuries, orthogeriatrics, the ambulatory care of minor trauma (with patients sent to the radiology department by a triage nurse), etc.

Methoxyflurane’s arrival on the market in France prompted us to apply the MCDA method in an ED context of pain management for the first time. In a pilot study, we sought to model the organizational impact of the introduction of MEOF for acute trauma pain in the ED, with and without the concomitant introduction of a trauma care pathway. We decided to apply a MCDA design process based on a multidisciplinary expert panel pilot study, in order to assess this novel method’s ability to model organizational impacts in the ED.

## Methods

The Rouen University Hospital Research Ethics Committee (Rouen, France) examined the protocol registered under file number E2019-66 and waived the requirement for ethics approval. Informed written consent was obtained for all participants.

## Objective

The objective of the present study was to ask a panel of professionals with significant expertise in the organization of integrated trauma care pathways to estimate the impact of using MEOF to treat moderate-to-severe acute trauma pain on several organizational criteria in the ED.

## Study procedures and participants

The study was designed and led by a specialist in medical decision-making [26]. The panel of experts was recruited by the Institute For Research And Studies In Health Organization (IREOS, Paris, France) between February and March 2018. Acute trauma pain management in the ED involves clinical and drug expertise. Accordingly, this study’s expert panel consisted of clinicians and researchers from different ED, and of Hospital Pharmacists participating in drug decision-making. In order to bring a diversity of perspectives for this pilot study, it was decided to recruit 8 experts, with a balanced representation of ED Physicians and Pharmacists, from 8 different urban tertiary hospitals. A first list of subject matter experts was identified through Pubmed literature search and via the professional network of the French Society of Emergency Medicine. Inclusion criteria comprised: (i) specialists in Emergency Medicine and pain management (for ED Physicians) or participation in decision-making committees, workgroups impacting policies and procedures affecting ED setting (for Pharmacists), (ii) healthcare professionals with significant expertise in the organization of integrated trauma care pathways, (iii) significant expertise in MEOF use and/or supply chain. No exclusion criteria were applied to participant recruitment. The experts were asked to participate as individual scientists, meaning they had to convey their own scientific opinions, not the views of the institutions they were affiliated with.

Twelve experts were contacted. Two declined, two did not respond, and eight accepted the invitation: four ED Physicians, all members of the French Society of Emergency Medicine (practicing at Rouen University Hospital, Grenoble University Hospital, Lyon Public Hospices, Avicenne-Bobigny Hospital, respectively) and four Pharmacists (Meaux Hospital, University Hospital of Bordeaux, Beaujon Hospital, Lyon Public Hospices, respectively).

**Fig 1:**
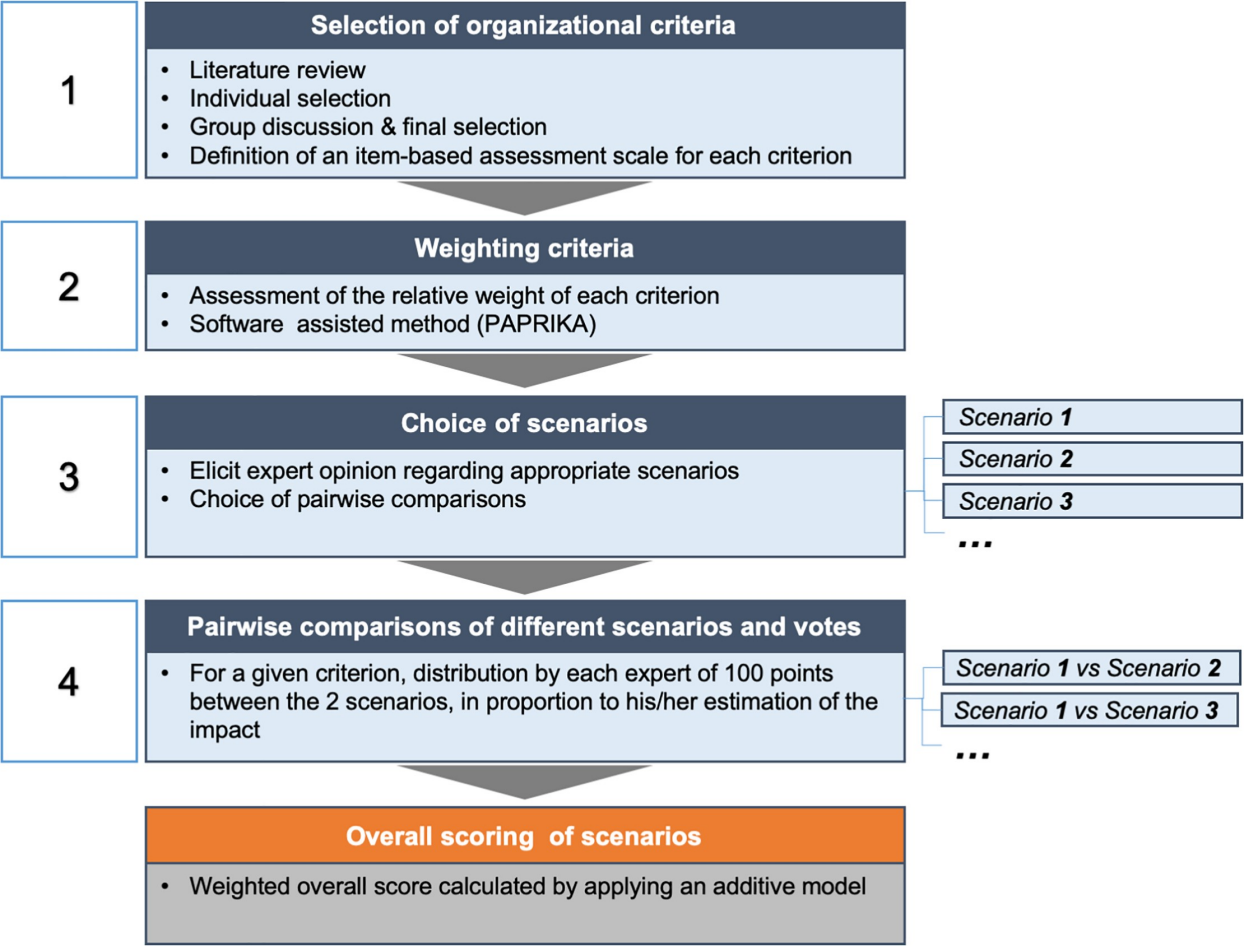
The MCDA process used to develop our specific model.

The research took place in Paris in May 2018. After a half-day briefing session on the specific MCDA approach that had been chosen, participants met for a 6-hour test session. The procedure comprised four steps (Fig 1): (i) selection of relevant criteria for measuring the organizational impact of the study intervention, (ii) weighting of the selected criteria, (iii) choice of appropriate scenarios for exploring the organizational impact of MEOX under various contexts, and (iv) estimation of the organizational impact of MEOF in pairwise comparisons of different scenarios (i.e. taking a trauma care pathway into account or not).

Numeric data for step (ii) (weighting of the selected criteria) and (iv) (estimation of the organizational impact of MEOF in pairwise comparisons of different scenarios) were developed using a conjoint-analysis methodology—the Potentially All Pairwise RanKings of all possible Alternatives (PAPRIKA) [27]. Conjoint analysis, or discrete choice experiment, is a statistical technique involving a simple ordinal–ranking–measurement of decision-makers’ preferences rather than a scaling or ratio measurement. As our aim was to produce consensual values rather than to compare individual data, we planned to focus our analysis to the calculated means, no complementary statistical analysis or test being applicable.

In the present pilot study, we considered that a sensitivity analysis would not be relevant in view of the panel’s selection procedure and the fact that the group discussion’s objective was to build a final consensus.

### Selection of the relevant criteria for evaluating the expected organizational impact

In the first part of the study, the experts studied a list of 542 possible criteria that had been extracted from an algorithmic literature analysis (S1 Table) and grouped into five organizational themes (as defined by the Danish Centre for Health Technology Assessment, National Board of Health [28]: 139 “Activity” criteria, 136”Environment” criteria, 109 “People” criteria, 71 “Structure” criteria, and 87 “Technology” criteria. From this list, each expert had to select the 8 criteria that he/she considered to be the most informative and then present them to the other experts, domain by domain. The objective of this group discussion was to form a consensus on the nature and number of selected evaluation criteria. For each criterion, an item-based assessment scale was defined by the expert group, in accordance with the guidelines [21,22].

### Weighting of the selected criteria

Given that criteria can have different relative weights with regard to the overall impact, we used a quantitative tool, and applied the PAPRIKA method [27] to determine the weightings in the second part of the study. This method compares pairs of criteria while considering that all the other criteria are identical. Firstly, PAPRIKA software (1000minds Ltd, Dunedin, New Zealand) was used to calculate the number of possible comparisons. Next, based on the number of comparisons and the number of experts, an algorithm was used to define a smaller set of discriminant choices and to mask the implicitly resolved comparisons. Once all the comparisons had been performed, the software calculated the weightings: from the answers, mathematical methods based on linear programming were used to calculate each expert preference values (or ‘part-worth utilities’), representing the relative importance (weights) of the criteria to her/him. This relative importance was expressed as a ratio (%) attributed to each criterion (100% representing the sum of the weights of all the criteria).

### Choice of scenarios

To achieve the study’s objective, the experts considered several alternative scenario comparisons in the third part of the study: before vs. after the introduction of the innovation, a “here vs. elsewhere” scenario (a comparison of the impact on institutions of different sizes and in different locations), MEOF vs. a reference analgesic, the presence vs. absence of comorbidities, etc. The final choice of the four scenarios was conditioned by three consensual high-priority factors: (i) the need for a trauma care pathway and standardized pain management, (ii) the pain relief strategy in the ED with regard to both the treatment (synergistic treatments, first-line treatments, follow-on treatments, etc.) and organizational aspects, and (iii) the presence of a trauma care pathway in an ED is more discriminant than the department’s size or patient volume.

The experts therefore chose to compare 4 alternative scenarios: Scenario 1 (S1): an ED without a trauma care pathway and without MEOF as a treatment option; S2: an ED without a trauma care pathway but with MEOF as a treatment option; S3, an ED with a trauma care pathway but without MEOF as a treatment option; S4, an ED with both a trauma care pathway and MEOF as a treatment option.

### Pairwise comparisons of scenarios

In the fourth part of the study, pairwise comparisons of these scenarios were performed, i.e. S1 vs. S2, S1 vs. S3, and S1 vs. S4. For each comparison, each expert had to estimate the impact on each of the selected criteria. For a given criterion, the expert had to distribute 100 points between the two scenarios, in proportion to his/her estimation of the impact. The impact was considered to be positive for the selected criterion if the expert attributed more than 50 points to the scenario.

After each expert had quantified the impacts, a weighted overall score was then calculated by applying an additive model. The score obtained with each of the three pairs of combinations showed whether the novel use of MEOF in acute trauma pain relief impacted one type of organization more than another. In the present pilot study, we considered that a sensitivity analysis would not be relevant in view of the panel’s selection procedure and the fact that the group discussion’s objective was to build a final consensus.

## Results

### Choice of the criteria for evaluating the organizational impact of MEOF

The expert group selected 9 criteria with their respective items for measurement: 3 in the “Activities” domain, 2 in the “Environment” domain, 2 in the “People” domain, 1 in the “Structure” domain and 1 in the “Technologies” domain. The criteria and the measurement scales for each criterion are presented in Table 1.

**Table 1:** Criteria for evaluation of the organizational impact and the respective items.

Field	Criteria	Measurement scale (items)
Activities	Waiting time before first treatment	Increased
		Neutral
		Reduced
	Mean length of stay in the emergency department	Increased
		Neutral
		Reduced
	Time before first analgesic delivery	>30 minutes
		<30 minutes
Environment	Standardization of the patient pathway	No
		Yes
	Hospital drug supply chain ^a^	Complicated
		Standard
People	Training and provision of information on patient pathways	Complicated
		Simplified
	Care workload	Not optimized
		Optimized
Structure	Acceptability of the innovation/strategy for patients/carers/patient associations/ healthcare professionals	Difficult
		Easy
Technologies	Facilities-related constraints ^b^	Yes
		No

^a^ Concerns the characteristics of the hospital drug supply chain, such as the specific conditions for storage, delivery, traceability, waste disposal, etc.

^b^ Concerns the possible logistic constraints in the ED service related to the product, such as storage space, a specific room for administration, etc.

### Comparisons and weightings of the criteria

**Fig 2:**
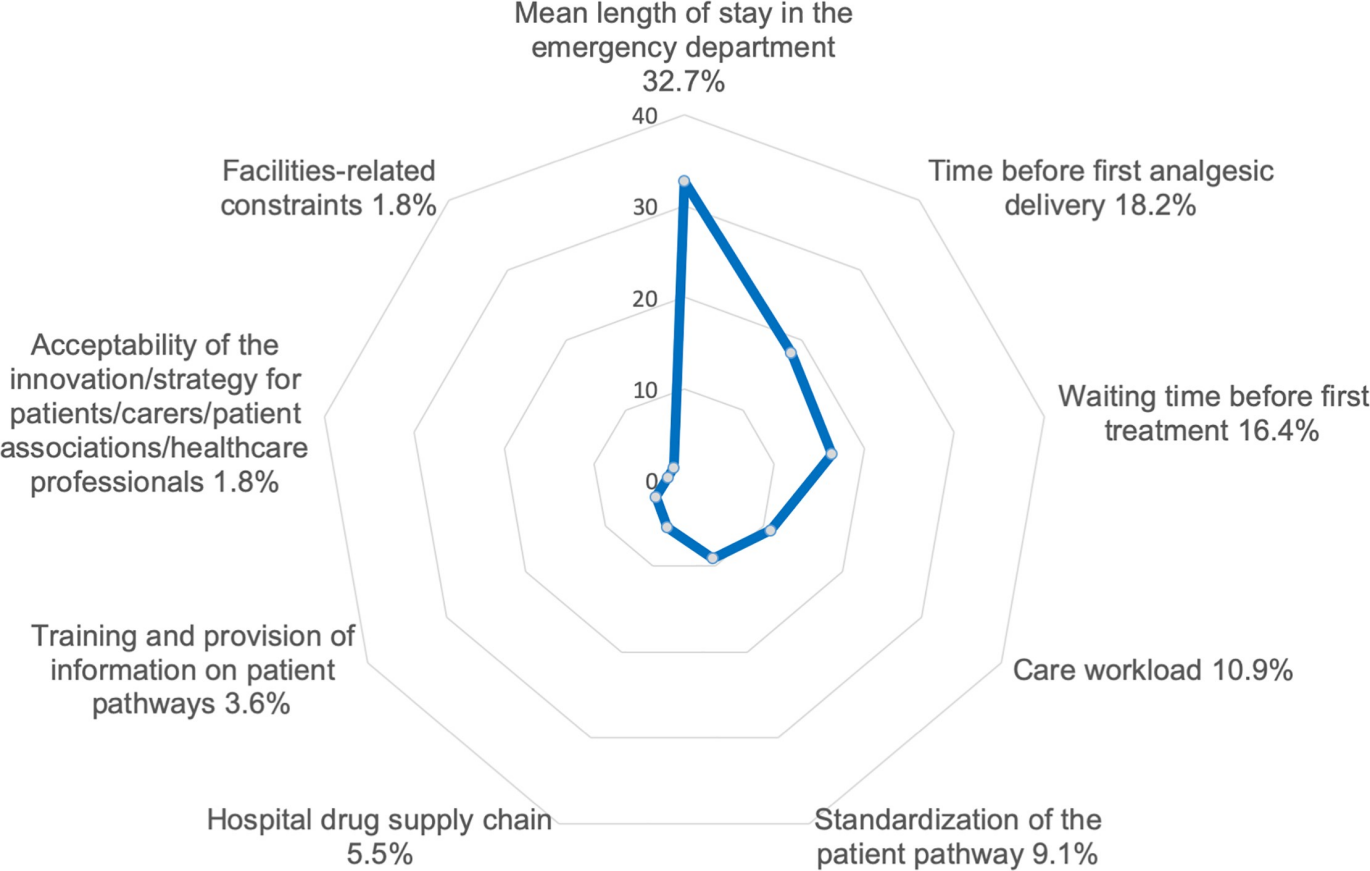
Weights attributed to each criterion.

In view of the large number of criteria and items selected, the PAPRIKA software detected 7,143 possible comparisons. Each expert then performed between 17 and 22 comparisons. The three most strongly weighted criteria were “Mean length of stay in the ED” (weight: 32.7%), “Time before first analgesic delivery” (18.2%), and “Waiting time before first treatment” (16.4%). The weights attributed to each criterion are shown in Fig 2.

### Assessment of the expected organizational impact

The introduction of MEOF in the absence of a trauma care pathway was rated by the experts as having a positive expected overall impact (S1 *vs*. S2; Table 2), with a total score of 59 out of 100. There was an expected positive impact on all the selected organizational criteria other than “Waiting time before first treatment” (neutral impact: 50 out of 100). The most frequently predicted (and greatest) positive expected impact was for “Time before first analgesic delivery” (70 out of 100).

**Table 2:** Assessment of the organizational impact for the first combination: Scenario 1 vs. scenario 2.

		Scenario 1 (no methoxyflurane and no trauma care pathway)		Scenario 2 (introduction of the methoxyflurane in the absence of a trauma care pathway)	
Criterion	Weight of the criterion (%)	Expected impact^1^	Weighted impact	Expected impact^1^	Weighted impact
Waiting time before first treatment	16.4	50	8.20	50	8.20
Mean length of stay in the ED	32.7	42	13.73	58	18.97
Time before first analgesic delivery	18.2	30	5.46	70	12.74
Standardization of the patient pathway	9.1	40	3.64	60	5.46
Hospital drug supply chain	5.5	40	2.20	60	3.30
Training and provision of information on patient pathways	3.6	40	1.44	60	2.16
Care workload	10.9	40	4.36	60	6.54
Acceptability of the innovation/strategy for patients/carers/patient associations/healthcare professionals	1.8	42	0.76	58	1.04
Facilities-related constraints	1.8	40	0.72	60	1.08
Total^2^	100	NA	40.51	NA	59.49

The expert group’s assessment of the organizational impact for each criterion, by comparing the introduction of MEOF in the absence of a trauma care pathway with the absence of MEOF and the absence of a trauma care pathway.

1. Expected impact: a score out of 100 was attributed by the experts for each scenario. The criterion was considered to have a positive impact if the expert gave a score of more than 50 out of 100 to one of the two scenarios.

2. Cumulative score.

ED: emergency department; NA: not applicable.

Implementation of a trauma care pathway alone (S1 *vs*. S3; Table 3) had a greater overall positive expected impact than MEOF alone, with a total score of 66 out of 100. It had a positive impact on 7 of the 9 organizational criteria, a neutral impact on the “acceptability of the innovation” criterion, and a negative impact on the “facilities-related constraints” criterion (45 out of 100).

**Table 3:** Assessment of the organizational impact for the second combination: Scenario 1 vs. scenario 3.

		Scenario 1 (no methoxyflurane and no trauma care pathway)		Scenario 3 (trauma care pathway but no methoxyflurane)	
Criterion	Weight of the criterion (%)	Expected impact^1^	Weighted impact	Expected impact^1^	Weighted impact
Waiting time before first treatment	16.4	30	4.92	70	11.48
Mean length of stay in the ED	32.7	30	9.81	70	22.89
Time before first analgesic delivery	18.2	40	7.28	60	10.92
Standardization of the patient pathway	9.1	30	2.73	70	6.37
Hospital drug supply chain	5.5	40	2.20	60	3.30
Training and provision of information on patient pathways	3.6	35	1.26	65	2.34
Care workload	10.9	40	4.36	60	6.54
Acceptability of the innovation/strategy for patients/carers/patient associations/healthcare professionals	1.8	50	0.90	50	0.90
Facilities-related constraints	1.8	55	0.99	45	0.81
Total^2^	100	NA	34.45	NA	65.55

The expert group’s assessment of the organizational impact for each criterion, by comparing the introduction of a trauma care pathway in the absence of MEOF with the absence of a trauma care pathway and the absence of MEOF.

1. Expected impact: a score out of 100 was attributed by the experts for each scenario. The criterion was considered to have a positive impact if the expert gave a score of more than 50 out of 100 to one of the two scenarios.

2. Cumulative score.

ED: emergency department; NA: not applicable.

When the MEOF was introduced together with a trauma care pathway (S1 *vs*. S4, Table 4), the overall positive expected impact was even greater (75 out of 100). We also observed positive expected impacts on all the individual criteria, and these impacts were greater than in the other scenarios.

**Table 4:** Assessment of the organizational impact for the third combination: Scenario 1 vs. scenario 4.

		Scenario 1 (no methoxyflurane and no trauma care pathway)		Scenario 4 (introduction of methoxyflurane and trauma care pathway)	
Criterion	Weight of the criterion (%)	Expected impact^1^	Weighted impact	Expected impact^1^	Weighted impact
Waiting time before first treatment	16.4	25	4.10	75	12.30
Mean length of stay in the ED	32.7	24	7.85	76	24.85
Time before first analgesic delivery	18.2	20	3.64	80	14.56
Standardization of the patient pathway	9.1	20	1.82	80	7.28
Hospital drug supply chain	5.5	30	1.65	70	3.85
Training and provision of information on patient pathways	3.6	28	1.01	72	2.59
Care workload	10.9	32	3.49	68	7.41
Acceptability of the innovation/strategy for patients/carers/patient associations/healthcare professionals	1.8	26	0.47	74	1.33
Facilities-related constraints	1.8	42	0.76	58	1.04
Total^2^	100	NA	24.78	NA	75.22

The expert group’s assessment of the organizational impact for each criterion, by comparing the concomitant introduction of MEOF and a trauma care pathway with the absence of MEOF and the absence of a trauma care pathway.

1. Expected impact: a score out of 100 was attributed by the experts for each scenario. The criterion was considered to have a positive impact if the expert gave a score of more than 50 out of 100 to one of the two scenarios.

2. Cumulative score.

ED: emergency department; NA: not applicable.

## Discussion

By performing a MCDA and determining the preferences of a multidisciplinary panel of expert physicians and pharmacists, we modelled the organizational impact of an innovation in acute trauma pain management in the ED.

The main finding of the present pilot study can be interpreted in the following manner: as an innovation in acute trauma pain relief, MEOF appears to have a positive impact on the majority of organizational criteria but has a smaller effect than the implementation of a trauma care pathway *per se* (i.e. care reorganization). One can hypothesize that the experts considered an institutional, “shared” protocol to be the most powerful means of fighting against individual resistance to the integration of a new tool into the ED’s therapeutic arsenal. Theoretical work on breakthrough innovations [29] mentions this form of cognitive resistance to change.

In reality, our modelling showed that MEOF can modulate and justify the implementation of a care pathway in the ED. The experts considered that the implementation of an innovation in pain management such as MEOF was intimately linked to the potential for care pathway implementation. This perception is in line with literature reports in which care pathway implementation was associated with improvements in pain management [30]. This finding also highlights the fact that MEOF has a unique administration route and procedure, and is innovative in several (mainly non-pharmacological) respects: a few inhalations provide effective relief, venous access is not required, and—above all—pain relief is based on self-administration by the patient. The treatment can thus be started without delay, as soon as the patient is admitted to the ED. Methoxyflurane is usable even when the ED is crowded and workloads are heavy. Given that the patient can self-treat his/her pain, the ED’s medical and paramedical staff have more time to devote to other tasks.

With regard to the nature of the organizational criteria highlighted here and their respective weights, several findings are especially noteworthy. Firstly, our results showed that the healthcare professionals’ opinions converged from the first step in the study onwards, as the weights attributed to certain criteria led to saturation. Secondly, given that “Mean length of stay in the ED” was a composite criterion, one might have expected it to be weakly discriminant or over-represented with a disproportionately high weight. However, when it was removed from the model, the total scores for each combination were almost all identical: the scores shifted from 59.5 to 60.2 for S1 vs. S2, from 65.5 to 63.2 for S1 vs. S3, and from 75.2 to 74.7 for S1 vs. S4.

In summary, our results highlighted the value of using a weighted MCDA process because it accurately reflected clinical reasoning, i.e. intuitive decision-making. Although there are other alternatives for multiple criteria decision support, MCDA is more easily applicable–as long as the number of scenarios is relatively small. Multiple criteria decision analysis protocols have already been tested successfully in other hospital services. The application of MCDA to emergency medicine is innovative, although comparisons of good practice guidelines with field-based observations in oncology have confirmed the relevance of this approach [31]. In the field of emergency medicine, the present study is the first to have highlighted organizational judgement criteria for ED physicians and hospital pharmacists. Some of the criteria were expected (on the basis of the literature data) but others are reported here for the first time.

Even though we adopted a well-proven method (MCDA) and selected our scenarios with parsimony, our pilot study suffered from the inherent limitations of modelling. Firstly, our study may have been limited by cognitive bias, due to each expert’s professional context (notably the type of practice) and his/her experience with MEOF. Secondly, certain sources of methodological bias could not be avoided, in view of our study’s multiple-criteria design. The total score only provides decision support by stimulating debate between experts and generating a consensus, after having revealed common trends. The panel’s size, composition and selection method prevented us from testing the experts’ preferences with a sufficient degree of precision. Larger studies of a more varied panel (including nurses and patients, for example) would enable this type of approach and this study design to be tested in a more relevant way (notably with support from robust quantitative analyses, such as sensitivity tests).

Thirdly, one can question (i) the relevance of some of the measurement scales (quantified items) that have not been validated in the field (a waiting time above or below 30 minutes, for example), (ii) the value of assessing a pain management strategy without taking account of the patient’s cognitive status, and (iii) the patient’s ability to manage self-administration of the drug in a truly independent way. Lastly, our simplification of the treatment scenarios is necessarily a limitation, relative to an *in situ* evaluation.

The value of predicted impact modelling should be compared with that of *a posteriori* evaluation (the evaluation of professional practice, lean management, etc.), once the treatment guidelines have been selected. We therefore plan to conduct a follow up study to test the results of our MCDA model and evaluate its reliability.

Upstream modelling probably helps to avoid fruitless investments—even in the short term—and limits dependency on single-center clinical studies with little scientific value. This type of work paves the way to implementation in a real organizational context, after the estimation of its impact in alternative scenarios. With regard to health economics, our hypotheses will have to be confirmed or rejected in conventional cost-benefit studies. However, for greater relevance, the hypotheses could target specific care pathways. Lastly, this approach could facilitate the relevant referencing of new treatments in the context of pain management protocols, while taking account of local resources.

## Conclusions/Perspectives

By performing a MCDA, we proposed a novel approach that was well suited to the context of an ED and that enabled us to assess the multi-criteria organizational impact of an innovation in pain management. Furthermore, the modelling method developed in this pilot study took account of an optimized trauma care pathway. When applied to the particular instance of MEOF, MCDA highlighted the positive organizational impact of the introduction of this therapeutic tool in the ED; our results argue in favor of the high-priority integration of MEOF into a specific care pathway for the management of acute trauma pain.

More generally, our present results confirmed the relevance of applying MCDA to emergency medicine and the establishment of an expert consensus in a complex decision-making process. Multiple criteria decision analysis processes take account of the large number of criteria–some of which are specific and unexpected–that should be considered by the main stakeholders (physicians, pharmacists, other healthcare professionals, patients, health decision-makers, etc.) when estimating the organizational impact of therapeutic innovations in the ED. The MCDA could probably be applied to fields of emergency medicine other than acute pain management if the problems faced are the same.

## Supporting information

S1 Table Algorithmic literature analysis used to determine criteria that experts have to consider for evaluating the expected organizational impact of methoxyflurane. (DOCX)
