# Supplementary material for: Multiple criteria decision analysis approach to consider therapeutic innovations in the emergency department: The methoxyflurane organizational impact in acute trauma pain
Source: PLoS One. 2020 Apr 15;15(4):e0231571. doi: 10.1371/journal.pone.0231571 (PMC7159203; doi:10.1371/journal.pone.0231571)
Supplement: S1 Table — (DOCX) [file pone.0231571.s001.docx]

**S1 Table.** **Algorithmic literature analysis used to determine criteria that experts have to consider for evaluating the expected organizational impact of methoxyflurane.**

|  | | **Search algorithms** |
| --- | --- | --- |
| “Criteria” | ((decision support tool[Title/Abstract]) AND ((HTA[Title/Abstract]) OR (health technology assessment[Title/ Abstract])) AND (criteria[Title/ Abstract])) OR ((decision support model[Title/ Abstract]) AND ((HTA[Title/ Abstract]) OR (health technology assessment[Title/Abstract])) AND (criteria[Title/Abstract])) OR ((decision making[Title/Abstract]) AND ((HTA[Title/Abstract]) OR (health tech no logy assessment[Title/Abstract])) AND (criteria [Title/Abstract])) OR ((decision making[MeSH Terms]) AND ( (HTA[Title/Abstract]) OR (health technology assessment[Title/Abstract])) AND (criteria [Title/Abstract])) OR ((decision support techniques[Title/Abstract]) AND ((HTA[Title/Abstract]) OR (health tech no logy assessment[Title/ Abstract])) AND (criteria[Title/Abstract])) | |
| “Systematic review” | ((decision support tool [Title/ Abstract]) AND ((HTA[Title/Abstract]) OR (health technology assessment[Title/ Abstract])) AND (systematic review[Title/Abstract])) OR ((decision support  model[Title/Abstract]) AND ((HTA[Title/Abstract]) OR (health technology assessment[Title/Abstract]))  AND (systematic review[Title/ Abstract])) OR ((decision making[Title/ Abstract]) AND ((HTA[Title/Abstract]) OR (health technology assessment[Title/Abstract])) AND (systematic review[Title/Abstract])) OR ((decision making[MeSH Terms]) AND ((HTA[Title/Abstract]) OR (health technology assessment[Title/ Abstract])) AND (systematic review[Title/ Abstract])) OR ((decision support techniques[Title/Abstract]) AND ((HTA[Title/ Abstract]) OR (health technology assessment[Title/Abstract])) AND (systematic review[Title/ Abstract])) | |
|  | |  |
